# Supplementary material for: Modeling the metabolic interplay between a parasitic worm and its bacterial endosymbiont allows the identification of novel drug targets
Source: eLife. 2020 Aug 11;9:e51850. doi: 10.7554/eLife.51850 (PMC7419141; doi:10.7554/eLife.51850)
Supplement: Supplementary file 1. [file elife-51850-supp1.docx]

# Modeling the metabolic interplay between a parasitic worm and its bacterial endosymbiont allows the identification of novel drug targets

David M. Curran, Alexandra Grote, Nirvana Nursimulu, Adam Geber, Denis Voronin, Drew R. Jones, Elodie Ghedin, and John Parkinson

# **Supplemental Information**

Contains:

Supplemental Table 1

Supplemental Table 2

Supplemental Table 3

|  | | Open | L3 | L3D6 | L3D9 | L4 | F30 | F42 | F120 | M30 | M42 | M120 |
| --- | --- | --- | --- | --- | --- | --- | --- | --- | --- | --- | --- | --- |
| Control | HOHG | 12.6 | 12.6 | 12.6 | 12.6 | 12.6 | 12.6 | 12.6 | 12.6 | 12.6 | 12.6 | 12.6 |
|  | HOLG | 12.6 | 12.6 | 12.6 | 12.6 | 12.6 | 12.6 | 12.6 | 12.6 | 12.6 | 12.6 | 12.6 |
|  | LOHG | 12.6 | 12.6 | 9.1 | 8.7 | 9.4 | 10.2 | 11.4 | 7.1 | 9.8 | 8.8 | 8.9 |
|  | LOLG | 10.2 | 7.2 | 7.2 | 6.4 | 6.5 | 7.0 | 7.6 | 6.5 | 7.3 | 6.7 | 6.4 |
| + pyruvate | HOHG | 12.6 | 12.6 | 12.6 | 12.6 | 12.6 | 12.6 | 12.6 | 12.6 | 12.6 | 12.6 | 12.6 |
|  | HOLG | 12.6 | 12.6 | 12.6 | 12.6 | 12.6 | 12.6 | 12.6 | 12.6 | 12.6 | 12.6 | 12.6 |
|  | LOHG | 12.6 | 12.6 | 10.3 | 10.2 | 10.7 | 11.3 | 12.6 | 8.3 | 11.2 | 10.5 | 12.1 |
|  | LOLG | 12.6 | 9.4 | 8.2 | 7.4 | 7.2 | 7.7 | 8.5 | 7.4 | 8.0 | 7.6 | 8.4 |

**Supplemental Table 1. The objective flux under a high *Wolbachia* weight.** This table compares the flux through the objective function with a *Wolbachia* weight of 1.0 in each life stage model under different nutrient conditions, with and without excess pyruvate.

|  | | Open | L3 | L3D6 | L3D9 | L4 | F30 | F42 | F120 | M30 | M42 | M120 |
| --- | --- | --- | --- | --- | --- | --- | --- | --- | --- | --- | --- | --- |
| Control | HOHG | 59.5 | 37.8 | 28.0 | 31.1 | 33.4 | 35.3 | 36.6 | 18.1 | 30.5 | 31.0 | 15.7 |
|  | HOLG | 48.5 | 37.1 | 24.4 | 24.5 | 30.8 | 27.4 | 27.3 | 17.0 | 26.2 | 25.7 | 15.7 |
|  | LOHG | 44.3 | 35.6 | 26.6 | 27.6 | 28.0 | 30.0 | 32.2 | 14.5 | 26.0 | 26.0 | 15.7 |
|  | LOLG | 24.1 | 23.4 | 22.9 | 23.0 | 22.8 | 21.8 | 23.2 | 13.5 | 21.0 | 21.0 | 15.7 |
| + pyruvate | HOHG | 69.1 | 37.8 | 31.0 | 31.9 | 33.5 | 37.2 | 37.5 | 21.2 | 31.1 | 32.6 | 15.7 |
|  | HOLG | 60.0 | 37.6 | 26.0 | 25.5 | 31.2 | 28.7 | 28.3 | 19.2 | 26.6 | 26.5 | 15.7 |
|  | LOHG | 54.3 | 37.5 | 28.6 | 29.6 | 29.0 | 33.4 | 34.2 | 18.1 | 28.3 | 29.1 | 15.7 |
|  | LOLG | 35.8 | 29.8 | 24.5 | 24.4 | 23.5 | 23.1 | 24.5 | 16.6 | 22.9 | 23.7 | 15.7 |

**Supplemental Table 2. The maximum objective flux achieved with varying *Wolbachia* weight.** This table compares the maximum flux through the objective function in each life stage model under different nutrient conditions, with and without excess pyruvate.

|  | Movement units (mean) | Movement units (standard deviation) |
| --- | --- | --- |
| Control | 18.54 | 2.11 |
| Fosmidomycin | 15.59 | 5.48 |
| MDL-29951 | 17.54 | 2.98 |
| Tenofovir | 21.23 | 1.68 |

**Supplemental Table 3. Motility of *B. malayi* was not affected by drug treatment.** Assessments were made on one female worm per well, with eight biological replicates per condition. A unique motility phenotype was observed with Fosmidomycin treatment, but results were not significant as measured using the Worminator assay.
